# Prevalence and associated factors of *Treponema pallidum* infection in a rural area of southwestern China

**DOI:** 10.1186/s12889-020-08952-7

**Published:** 2020-06-01

**Authors:** Ying Shi, Ya Yang, Yingjian Wang, Dongjian Yang, Yu Yang, Shurong Dong, Chunlin Li, Yue Chen, Qingwu Jiang, Yibiao Zhou

**Affiliations:** 1 Fudan University School of Public Health, Building 8, 130 Dong’an Road, Xuhui District, Shanghai, 200032 China; 2 Key Laboratory of Public Health Safety, Fudan University, Ministry of Education, Building 8, 130 Dong’an Road, Xuhui District, Shanghai, 200032 China; 3 Fudan University Center for Tropical Disease Research, Building 8, 130 Dong’an Road, Xuhui District, Shanghai, 200032 China; 4 School of Epidemiology and Public Health, Faculty of Medicine, University of Ottawa, 451 Smyth Road, Ottawa, Ontario K1H 8M5 Canada

**Keywords:** *Treponema pallidum*, Syphilis, Risk factors, China

## Abstract

**Background:**

Epidemiological data on *Treponema pallidum* infection are scarce from the southwestern region of China. The purpose of this study was to determine the distribution and determinants of *T. pallidum* infection in the region.

**Methods:**

A community-based cross-sectional study of 2608 participants aged ≥14 years was conducted in a rural area of southwestern China in 2014–15. A pretested questionnaire was used to collect sociodemographic characteristics and other factors associated with *T. pallidum* infection. The diagnoses of *T. pallidum*, human immunodeficiency virus (HIV), hepatitis B virus (HBV) and hepatitis C virus (HCV) infections were determined by commercial test kits. Logistic regression analysis was used to determine the correlates for *T. pallidum* infection, and adjusted odds ratios (aORs) and 95% confidence intervals (CIs) were calculated.

**Results:**

The prevalence of *T. pallidum* infection was 1.2% (95% CI 0.8 to 1.7%). Risk factors varied by gender. In the male group, *T. pallidum* infection was significantly associated with ever injection drug use (aOR = 9.42, 95% CI 2.47 to 35.87) and HCV infection (aOR = 13.28, 95% CI 3.20 to 51.70). In the female group, correlates for *T. pallidum* infection included spouse having syphilis (aOR = 126.66, 95% CI 7.58 to 2122.94), ever having blood transfusion (aOR = 10.51, 95% CI 1.58 to 41.21) and HBV infection (aOR = 4.19, 95% CI 1.35 to 10.93).

**Conclusions:**

The prevalence of *T. pallidum* infection was high in the rural area of southwestern China. Correlates for *T. pallidum* infection varied with sex specific. Intervention should be developed for the prevention and control of *T. pallidum* infection.

## Background

Syphilis is a sexually and vertically transmitted disease (STD), which is caused by the *spirochaete Treponema pallidum* subspecies pallidum [1]. The World Health Organization (WHO) estimated that 17.7 million individuals aged 15–49 years had syphilis globally in 2012, with 5.6 million new cases every year [2]. In the low and middle-income countries, the risk for heterosexual spread of syphilis has declined in the general population but remains a challenge in some high-risk subpopulations, such as female sex workers (FSWs) and their male clients [1]. The infection of *T. pallidum* can cause cutaneous lesions, late complications such as neurologic and cardiovascular disease [3, 4], and congenital syphilis with other immediate complications, such as premature labor and low birthweight [5].

Syphilis was first recognized in mainland China in 1505 [6]. In 1949, the country experienced one of the biggest syphilis epidemics in human history [6]. The prevalence was 84% in prostitutes compared to 5% in general population in metropolitan area, and 2–3% in less developed cities in the 1950s [6]. Chinese government lunched an unprecedented campaign to make syphilis eradicated in 1960s [7]. However, syphilis has revived in China since the reform and opening up, and the prevalence raised about 5% among FSWs and 3% among their male clients [1]. Most of previous studies were executed among high-risk groups such as FSWs, IDUs, men who have sex with men, while few studies were carried out in general population.

Epidemiological data on syphilis prevalence is critical for developing public health strategies towards syphilis prevention, care and treatment. Our study site is one of the largest illicit drug distribution centers of China, and the largest autonomous prefecture of Yi nationality. Frequent casual sexual behavior is more tolerable among Yi people, which is related to an increased risk of *T. pallidum* infection [8]. In this region there are few female commercial sex workers and men who have sex with men among the Yi people [9]. In the current report, we aimed to determine the prevalence and factors associated with *T. pallidum* infection among local residents, mainly Yi people, in this region.

## Methods

### Study site and population

A population-based cross-sectional study was conducted from October 2014 to August 2015, in a Yi Prefecture of southwestern China, where almost half the residents are of the Yi ethnicity. It remains one of the most undeveloped regions in China as a result of mountainous terrains coupled with sparsely distributed population. A two-stage sampling was conducted in the study. Three counties (Pg, Zj, and Mg) were selected in first step, whose residents shared similar health and socio-demographic characteristics, such as the proportion of minority, ethnic identity, age structure, and education level. Subsequently, four towns were selected from these counties by simple random sampling, where A and B from Pg County, C from Zj county and D from Mg county. Residents were eligible for investigation if they had to be over 14 years old and lived here for more than 6 months. The individual over14 years is permitted to have sexual practices, one of the criteria attributed to local Yi culture. We invited those who met the enrollment criteria to participate in our study. Village chiefs were asked to coordinate the side-by-side observations and the communication between local dialects and official language.

### Data collection

Well trained local health professionals were responsible for questionnaire interview and screening test. All participants were informed of the objectives, contents, potential risk and benefits of this survey prior to the data collection. Study participants were assigned a unique identifier number to collect data confidentially. Face-to-face interviews were conducted and a structured questionnaire (see Additional file 1) covered sociodemographic characteristics (including age, sex, ethnicity, marital status, education, occupation, and household annual income), risky sexual behaviors (frequency of condom use and having multiple sexual partners), drug abuse and history of blood transfusion.

### Blood sample collection and diagnosis

All participants were screened for *T. pallidum* antibody using the Syphilis Antibody Rapid Test (Colloidal Gold) (Acon Biotech Co. Ltd., Hangzhou, China), which has a sensitivity of 99.5% and a specificity of 99.0%. Anti-HIV antibodies were tested with the Diagnostic Kit for HIV (Colloidal Gold) (Livzon Pharmaceutical Group Inc., Zhuhai, China). Each participant had a finger prick and provided about 1 mL of blood by using the Diagnostic Kit for HBV surface antigen (Colloidal Gold) (product of Livzon Pharmaceutical Group Inc., Zhuhai, China), together with HCV antibody (Colloidal Gold) (product of Livzon Pharmaceutical Group Inc., Zhuhai, China). Product specifications show that the sensitivity and specificity of the colloidal gold kits are both higher than 95%. Immunochromatographic assay strips are simple, inexpensive, rapid, robust and equipment free, which have been widely used for sexually transmitted diseases screening in many resource-constrained settings [10]. All strips were within the period of validity indicated by the manufacturer. Screening procedures were carried out strictly according to the manufacturer’s instructions. We collected a 5 mL blood sample from those with a positive result, immediately transported it to local township hospitals. Whole blood was centrifuged at 1000 rpm for 5 min and plasma was separated and stored at the constant temperature of − 20 °C within 8 h, and then were transported in ice to Shanghai. Plasma aliquots were tested for viral load by real-time polymerase chain reaction (PCR). The application of real-time PCR assays with a high specificity (98.0%) for secondary syphilis can detect the pathogen directly, and avoid the modification of HIV status [11–13]. Also, there was no further need to consider the syphilis serological window period [13]. We applied the diagnostic kit for the quantification of *T. pallidum* DNA, HBV DNA, HCV RNA, and HIV-1 RNA (PCR-Fluorescence Probing) (DaAn Gene Group Inc., Zhongshan, China) for Plasma viral loads in the Center for Tropical Disease Research at Fudan University. HCV RNA in serum was detected for the majority (95%) of patients with antibodies [14].

### Ethical considerations

Written informed consents were obtained from all participants. They received the results of the rapid testing on the same day. Those with positive results were provided with appropriate medical consultations, further examinations, and treatments. The procedures of this study were reviewed and approved by the Ethical Review Committee of School of Public Health, Fudan University.

### Statistical analysis

Data entry employed the EpiData software (version 3.1; the EpiData Association, Odense, Denmark). The data processing and analysis was performed with the SPSS software (version 18.0, IBM SPSS Institute, Inc., Chicago). Descriptive statistics were carried out for sociodemographic variables. The proportions of *T. pallidum*, HBV, HCV and HIV infections and co-infections were calculated as well as their 95% confidence intervals (CIs). Pearson χ^2^ test or Fisher’s exact test was applied to examine potential variables associated with *T. pallidum* infection. Crude odds ratios (cORs) along with their 95% CIs were also calculated. A stepwise logistic regression was employed to build a final multivariable model (*P* value of retention≤0.05). Adjusted ORs and 95% CIs were calculated for identified associated factors. Since sex was interacted with some covariates, sex-specific analysis was performed. The statistical significance was symbolized by a two-sided *P* value ≤0.05.

Quality controlling procedures were followed throughout the process by trained personnel. Skilled local health professionals with medical degrees were employed for blood sample collection and diagnosis at township health clinics. Data accuracy were routinely checked through cross-tabulations and logical checks, Double data entry was taken for data entry quality control.

## Results

A total of 2608 participants over 14 years underwent *T. pallidum,* HBV, HCV, HIV screening tests and completed a questionnaire interview. Demographic characteristics of the participants were summarized in Table 1. Most of them were farmers (91.3%), ever worked away from home (83.0%) and were Yi people (96.6%). Majority of them were female (65.2%), married (83.6%) and illiterate (64.7%).

**Table 1:** Demographic characteristics of 2608 participants aged ≥14 years

Characteristics	No. (percentage)
**Town**	
A	524 (20.1%)
B	571 (21.9%)
C	968 (37.1%)
D	545 (20.9%)
**Sex**	
Female	1700 (65.2%)
Male	908 (34.8%)
**Age group (years)**	
14–24	413 (15.8%)
25–34	592 (22.7%)
35–44	772 (29.6%)
45–54	558 (21.4%)
≥ 55	273 (10.5%)
**Ethnicity**	
Yi	2519 (96.6%)
Han and others	89 (3.4%)
**Education**	
Illiteracy	1676 (64.3%)
Primary school	655 (25.1%)
High school and above	277 (10.6%)
**Occupation**	
Farmers	2382 (91.3%)
Students	150 (5.8%)
Others	54 (2.1%)
'Missing’	22 (0.8%)
**Ever working away from home**	
No	442 (17.0%)
Yes	2166 (83.0%)
**Annual income**	
< 1000	407 (15.6%)
1000–2999	922 (35.4%)
3000–4999	603 (23.1%)
5000–9999	321 (12.3%)
≥ 10,000	331 (12.7%)
'Missing’	24 (0.9%)
**Marital status**	
Married	2181 (83.6%)
Unmarried	427 (16.4%)

Among them, 1.2% (95% CI 0.8 to 1.7) were infected with *T. pallidum*, 5.6% (95% CI 4.7 to 6.5) with HIV, 7.4% (95% CI 6.5 to 8.5) with HBV and 2.7% (95% CI 2.1 to 3.4) with HCV. Only 0.1% (95% CI 0 to 0.4) were co-infected with HIV and *T. pallidum*, 0.2% (95% CI 0 to 0.5) with HBV and *T. pallidum*, and 0.2% (95% CI 0 to 0.5) with HCV and *T. pallidum* (Table 2).

**Table 2:** Prevalence of *T. pallidum*, HIV, HBV and HCV infections and coinfections

	No. Cases	Prevalence (%)	95%CI
*T. pallidum*	32	1.2	0.8–1.7
HIV	145	5.6	4.7–6.5
HBV	194	7.4	6.5–8.5
HCV	71	2.7	2.1–3.4
Co-infected with HIV and *T. pallidum*	4	0.1	0–0.4
Co-infected with HBV and *T. pallidum*	6	0.2	0–0.5
Co-infected with HCV and *T. pallidum*	6	0.2	0–0.5

Table 3 shows the correlates associated with *T. pallidum* infection in males and females combined. They were spouse having syphilis (aOR = 38.51, 95% CI 7.12 to 208.16), injection drug use (aOR = 3.48, 95% CI 1.14 to 10.57), blood transfusion (aOR = 5.70, 95% CI 1.12 to 28.97), HBV infection (aOR = 2.76, 95% CI 1.00 to 6.92), and HCV infection (aOR = 8.34, 95% 3.01 to 19.84).

**Table 3:** Univariate and multivariate analyses of factors associated with syphilis infection

Variables	No.	Cases (%)	cOR (95% CI)	aOR (95% CI)
**Town**				
A	524	9 (1.7)	Ref.	
B	571	7 (1.2)	0.71 (0.25–1.96)	
C	968	12 (1.2)	0.72 (0.30–1.78)	
D	545	4 (0.7)	0.43 (0.11–1.36)	
**Sex**				
Female	1700	22 (1.3)	Ref.	
Male	908	10 (1.1)	0.86 (0.38–1.78)	
**Age group (years)**				
14–24	413	3 (0.7)	Ref.	
25–34	592	9 (1.5)	2.04 (0.59–9.71)	
35–44	772	14 (1.8)	2.42 (0.78–11.03)	
45–54	558	4 (0.7)	0.97 (0.20–5.32)	
≥ 55	273	2 (0.7)	1.03 (0.12–6.82)	
**Ethnicity**				
Yi	2519	32 (1.3)		
Han and others	89	0 (0.00)		
**Education**				
Illiteracy	1676	25 (1.5)	Ref.	
Primary school	655	4 (0.6)	0.42 (0.12–1.09)	
High school and above	277	3 (1.1)	0.76 (0.17–2.19)	
**Occupation**				
Students	150	0 (0.00)	–	
Farmers	2382	31 (1.3)	0.70(0.09–5.24)	
Others	54	1 (1.9)	Ref.	
**Ever working away from home**				
No	442	5 (1.1)	Ref.	
Yes	2166	27 (1.2)	1.08 (0.45–3.24)	
**Annual income**				
< 1000	407	2 (0.5)	Ref.	
1000–2999	922	13 (1.4)	2.72 (0.74–19.00)	
3000–4999	603	9 (1.5)	2.90 (0.73–20.91)	
5000–9999	321	5 (1.6)	3.06 (0.63–23.93)	
≥ 10,000	331	3 (0.9)	1.81 (0.27–15.64)	
**Marital status**				
Ever married	2181	31 (1.4)	Ref.	
Never married	427	1 (0.2)	6.14(0.84–45.12)	
**Spouse syphilis**				
No or unknown	2601	30 (1.2)	Ref.	Ref.
Yes	7	2 (28.6)	35.39 (4.42–180.06) ^**^	38.51 (7.12–208.16) ^**^
**Ever using injection drugs**				
No	2519	28 (1.1)	Ref.	Ref.
Yes	89	4 (4.5)	4.32 (1.23–11.39) ^*^	3.48 (1.14–10.57) ^*^
**Having multiple sexual partners**				
No	2181	22 (1.0)	Ref.	
Yes	427	10 (2.3)	2.37 (1.06–4.95) ^*^	
**Condom use**				
Always or occasionally	562	12 (2.1)	Ref.	
Never	1442	14 (1.0)	0.45 (0.20–1.00) ^*^	
**Ever having blood transfusion**				
No or unknown	2580	30 (1.2)	Ref.	Ref.
Yes	28	2 (7.1)	6.96 (1.00–24.98) ^*^	5.70 (1.12–28.97) ^*^
**HIV infection**				
No	2463	28 (1.1)	Ref.	
Yes	145	4 (2.8)	2.55 (0.73–6.64)	
**HBV infection**				
No	2412	26 (1.1)	Ref.	Ref.
Yes	194	6 (3.1)	2.99 (1.09–6.92) ^*^	2.76 (1.00–6.46) ^*^
**HCV infection**				
No	2485	26 (1.0)	Ref.	Ref.
Yes	71	6 (8.5)	8.87 (3.17–21.11) ^**^	8.34 (3.01–19.84) ^**^

^*^P < 0.05, ^**^P < 0.01

Table 4 shows the results of sex-specific analysis. Injection drug use (aOR = 9.42, 95% CI 2.47 to 35.87) and HCV infection (aOR = 13.28, 95% CI 3.20 to 51.70) were related to *T. pallidum* infection in males. Spouse having syphilis (aOR = 126.66, 95%CI 7.58 to 2122.94), blood transfution (aOR = 10.51, 95% CI 1.58 to 41.21), and HBV infection (aOR = 4.19, 95% CI 1.35 to 10.93) were significantly associated with syphilis in females.

**Table 4:** Sex-specific univariate and multivariate analyses of factors associated with syphilis infection

Variables	Male (***N*** = 608)			Female (***N*** = 1700)		
	Prevalence (%)	cOR (95% CI)	aOR (95% CI)	Prevalence (%)	cOR (95% CI)	aOR (95% CI)
**Age group (years)**						
14–24	0	-		1.3	Ref.	
25–34	1.0	0.95(0.09–10.59)		1.8	1.34 (0.36–6.64)	
35–44	2.2	2.01(0.24–16.90)		1.6	1.23 (0.34–5.96)	
45–54	0.6	0.65(0.03–8.46)		0.8	0.61 (0.10–3.58)	
≥ 55	1.1	Ref.		0.5	0.47 (0.02–4.07)	
**Ethnicity**						
Yi	0			0		
Han and others	1.2			1.3		
**Education**						
Illiteracy	2.3	Ref.		1.2	Ref.	
Primary school	0	–		1.4	1.13 (0.31–3.13)	
High school and above	0.6	0.25(0.03–2.00)		1.8	1.56 (0.23–5.64)	
**Occupation**						
Students	0			0	–	
Farmers	1.1			1.2	0.36(0.05–2.81)	
Others	0			0.1	Ref.	
**Ever working away from home**						
No	0.6	0.59 (0.02–3.25)		1.4	Ref.	
Yes	1.2	Ref.		1.3	0.88 (0.32–3.14)	
**Annual income**						
< 1000	0			0.7	Ref.	
1000–2999	1.0			1.6	2.07 (0.53–14.82)	
3000–4999	2.6			1.0	1.30 (0.24–10.57)	
5000–9999	1.5			1.1	2.15 (0.32–18.66)	
≥ 10,000	0			1.1	2.15 (0.32–18.66)	
**Marital status**						
Ever married	1.4			1.4	Ref.	
Never married	0			0.4	0.32 (0.01–1.52)	
**Spouse syphilis**						
No or unknown	1.0	Ref.		1.2	Ref.	Ref.
Yes	20.0	26.54 (0.91–217.16)		50.0	78.04 (1.96–3015.11) ^**^	126.66 (7.58–2122.94) ^**^
**Ever using injection drugs**						
No	0.7	Ref.	Ref.	1.3	–	
Yes	5.4	7.94 (1.91–29.32) ^**^	9.42 (2.47–35.87) ^**^	0	–	
**Having multiple sexual partners**						
No	0.8	Ref.		1.1	Ref.	
Yes	2.1	2.55 (0.62–9.27)		2.6	2.41 (0.84–5.98)	
**Condom use**						
Always or occasionally	1.5	Ref.		2.5	Ref.	
Never	1.2	0.79 (0.20–3.98)		0.8	0.34 (0.12–0.90)	
**Ever having blood transfusion**						
No or unknown	1.1			1.2	Ref.	Ref.
Yes	0			10.5	10.32 (1.43–39.98) ^*^	10.51 (1.58–41.21) ^**^
**HIV infection**						
No	1.0	Ref.		1.2	Ref.	
Yes	2.5	2.73 (0.37–11.42)		3.1	2.78 (0.40–9.89)	
**HBV infection**						
No	1.1	Ref.		1.1	Ref.	Ref.
Yes	1.3	1.3 (0.06–7.68)		4.2	4.11 (1.31–10.71) ^*^	4.19 (1.35–10.93) ^**^
**HCV infection**						
No	0.7	Ref.	Ref.	1.2	Ref.	
Yes	7.4	11.18 (2.67–41.63) ^**^	13.28 (3.20–51.70) ^**^	11.8	11.42 (1.58–44.99) ^*^	8.93 (0.82–96.97)

^*^P < 0.05, ^**^P < 0.01

## Discussion

Overall, 1.2% (95% CI 0.8 to 1.7%) of the study population were infected with *T. pallidum* in this study, which was markedly higher than the estimated prevalence for the 15–59-year group in China (0.02%) [2]. A high proportion of ever using injection drugs (3.41%) is likely a main reason for this increased risk of *T. pallidum* infection in the region.

The correlates varied with sex due to different behavioral characteristics. There is a ‘Golden Triangle’ in the vicinity of our study sites, where a great number of illicit substances are produced and traded, resulting in a serious epidemic of drug abuse. Males exhibit higher rates of drug abuse and addiction than females [15]. Drug abuse was more common in males (8.3%) than females (0.9%) in our study. The drug culture of Yi ethnicity was shaped by male masculinity and peer solidarity. Injection and needle sharing were social activities that took place in a collective context [16]. Even in drug users, women only shared needles with their sexual partners, while in male injectors, needle sharing were more likely to occur with peer activities. Intravenous drug users (IDUs) had a higher prevalence of syphilis, which is consistent with the results from previous studies [17–19]. *T. pallidum*, HIV, HCV and HBV infections share common modes of transmission, leading to co-infections [20, 21]. Syphilis could generate genital ulcers and increase the likelihood of HIV virus shedding [21, 22]. The course of syphilis is accelerated in patients infected with HIV, and these patients frequently exhibit atypical lesions [23, 24].

These pathogens differ in their transmission efficiency with exposure types [25]. Previous studies showed that HCV and *T. pallidum* transmissions were more efficient than HIV transmission [26, 27]. Our study illuminated that HCV infection, but not HIV infection, was significantly associated *T. pallidum* infection in men. *T. pallidum* infection might contribute to HCV/HIV coinfection or HCV infection [26]. A study in FSWs shows that HCV is spread mainly by blood, rarely by sexual contact [27], which could explain why HCV infection associated with *T. pallidum* infection only occurred in males. Although HBV infection is not a classical STD, it can be transmitted through sexual contact with low efficiency [28].

In our study, 30 out of the 89 subjects who used drugs had a co-infection. These results highlighted the necessity of routine HCV testing for *T. pallidum* infected individuals [29], and also suggested that preventing syphilis should be included as a part of HBV control strategies. Targeted trainings for physicians and expanded syphilis screening services for HBV positive individuals are urgently needed [30, 31].

Consistent with previous studies, spouse having syphilis was a risk factor for *T. pallidum* infection in females [32] while syphilis was associated with drug abuse in males. Sharing syringes and drug related sexual activities dramatically increase the risk of *T. pallidum* infection [33, 34]. IDUs who engage in unprotected sex, place their female spouses at high risk for STDs [35]. It is common for Yi drug users to engage in sexual behaviors with no condom [36], and the proportion of condom use is extremely low [37]. Therefore, females are highly vulnerable to *T. pallidum* infection due to their unprotected sexual contacts with their high-risk husbands.

Blood transfusion and HBV infection were correlated with *T. pallidum* infection in females. Females have a higher probability to have a blood transfusion. Blood transfusion represents 5.8% of admissions for birth [38]. There is a great percentage of surgically treated females than males who are blood transfused [39]. Syphilis may facilitate an acquisition of HBV infection because of its ulcerative infection [40]. Lower socioeconomic status of local females may also be a factor [41]. HBV infection is endemic in China, and HBV vaccination rates are lower in females than males partly due to patriarchal tradition [42].

*T. pallidum* could be transmitted from mother to child in utero [43]. More than half of pregnant women with syphilis have a spontaneous abortion or stillbirth [44]. Inadequately treated *T. pallidum* infection can result in transplacental infection of the fetus, causing perinatal death, prematurity, low birth weight, congenital anomalies or long-term sequelae including deafness and neurological impairment [45]. Improved access to quality antenatal care, including rapid *T. pallidum* testing and latent syphilis treatment, is important for achieving the elimination of congenital syphilis [43, 46]. Policy-makers may expand and fund programs more effectively in future as one part of the broader harm reduction strategy under the China’s national HIV/STD control and prevention plan [18]. The Chinese government has also committed to eliminate the mother-to-child transmissions of HIV, syphilis and HBV with an ambitious plan for prenatal screening being executed nationwide. Given the high rate of co-infection with HCV found in this study area, pregnant women should also be screened for HCV.

Male IDUs present a greater risk of *T. pallidum* transmission to women. Risk reduction programs, such as condoms promotion, Maintenance Therapy programs (MMT) and Needle or Syringe Exchange Programs (NSEPs) should take into account for distinguishing behavior feature between males and females [9]. Education programs should be sensitive to the whole IDU community and emphasize the importance of consistent condom use with their sexual partners.

There are several limitations in this study. There might exist false-negative testing results because of the sensitivity of the colloidal gold kits. Our estimate for the prevalence of *T. pallidum* infection could be conservative, because many young individuals, who had a higher risk of *T. pallidum* infection, were more likely to search jobs outside and were not able to participate in the study. In addition, the history of drug abuse and other high-risk behaviors relied on self-reporting without further verification, which might result in a misclassification bias for the estimation of associations between study factors and *T. pallidum* infection. The number of syphilis-infected people was small and there might be a lack of statistical power to detect certain associations. The cross-sectional design did not allow us to assume any causal relationships between associated factors and *T. pallidum* infection.

## Conclusions

The risk of *T. pallidum* infection was high in the rural area of southwestern China, and was significantly associated with spouse having syphilis, drug abuse, blood transfusion and HBV and HCV infections. The correlates of *T. pallidum* infection varied in males and females. It is vital to implement comprehensive and effective intervention programs to reduce the risk of *T. pallidum* transmission and to achieve adequate access to syphilis treatment, especially for people who are IDUs and their spouses.

## Supplementary information

**Additional file 1.** The questionnaire on behaviors related to infectious diseases among people in Liangshan, A structured questionnaire in English.

## Data Availability

The datasets used and/or analyzed during the current study are available from the corresponding author on reasonable request.

## Abbreviations

aORs: Adjusted odds ratios
CIs: Confidence Intervals
cORs: Crude odds ratios
FSWs: Female Sex Workers
HBV: Hepatitis B Virus
HCV: Hepatitis C Virus
HIV: Human Immunodeficiency Virus
IDUs: Intravenous Drug Users
MMT: Maintenance Therapy Programs
NSEPs: Needle or Syringe Exchange Programs
PCR: Polymerase Chain Reaction
STD: Sexually Transmitted Disease
*T. pallidum*: *Treponema pallidum*
WHO: World Health Organization
