# Supplementary material for: Prevalence and associated factors of Treponema pallidum infection in a rural area of southwestern China
Source: BMC Public Health. 2020 Jun 1;20:824. doi: 10.1186/s12889-020-08952-7 (PMC7268706; doi:10.1186/s12889-020-08952-7)
Supplement: Supplementary file 1 — Additional file 1. The questionnaire on behaviors related to infectious diseases among people in Liangshan, A structured questionnaire in English. [file 12889_2020_8952_MOESM1_ESM.docx]

**The questionnaire on behaviors related to infectious diseases among people in Liangshan**

Individual Number: Head of a household:

1. General condition
2. Name:
3. Sex: ① Male ② Female
4. ID card No.:
5. Current address:
6. Date of birth:
7. Ethnicity: ① Han ② Yi ③ Others.
8. Marital status: ① Unmarried ② Newly married ③Remarried: times: ④ Widowed
9. Education：① Illiteracy ② Primary school ③ Middle school ④ Senior high school/ Technical secondary school/ Vocational High School ⑤ Junior college/ University and above
10. Occupation: ① Farmers ② Workers ③ Staff ④ Entrepreneur ⑤ Others:
11. Have you ever working away from home in the past three years？
    1. No ② Yes, less than 1year. ③ Yes, more than 1 year.
12. Annual household income: ① ＜1000 yuan ② 1000-3000 yuan ③ 3000-5000 yuan ④ 5000-10000 yuan ⑤ 10000-30000 yuan ⑥ >30000 yuan
13. Related behaviors
14. Do you ever have a boyfriend (girlfriend)? ① Yes， once had boyfriend (girlfriend). ② No.
15. Do you ever have sex? ① Yes, the first time happened when you were years old. ② No.
16. Do you know about the condoms? ① Yes. ② No.

14.1 Do you use condoms when having sex with a spouse? ① Often ② Once in a while ③ Never.

14.2 Do you use condoms when having sex with a boyfriend (girlfriend)? ① Often ② Once in a while ③ Never.

1. Have you ever suffered from drug addiction? ① Yes. The first time is ② No. (skip to 18)
2. In the last six months, Ways of taking drugs: ① Only oral taking or snorting ② Only injection ③ Mixing injection and other methods
3. In the last six months, have you ever shared syringes? ① Yes, shared with persons. ② No.
4. Have you ever received blood transfusion? ① Yes, reason for blood transfusion is (specific disease/accident). ② No.
5. The condition of spouses
6. Does your spouse suffer from drug abuse? ① Yes. ② Once, and detoxified now. ③ No.
7. Does your spouse infect with HIV? ① Yes. The diagnosis time is ② No. ③ I don’t know.
8. Does your spouse infect with HBV? ① Yes. The diagnosis time is ② No. ③ I don’t know.
9. Does your spouse infect with HCV? ① Yes. The diagnosis time is ② No. ③ I don’t know.
10. Does your spouse infect with syphilis? ① Yes. The diagnosis time is ② No. ③ I don’t know.
11. Results of tests
12. The rapid test of HIV: ① Positive (+) ② Negative(-)
13. The rapid test of HBV: ① Positive (+) ② Negative(-)
14. The rapid test of HCV: ① Positive (+) ② Negative(-)
15. The rapid test of syphilis: ① Positive (+) ② Negative(-)

Your contact information/telephone number:

Investigator: Date:
